# Hypomethylation in the promoter region of *ZPBP* as a potential litter size indicator in Berkshire pigs

**DOI:** 10.5194/aab-62-69-2019

**Published:** 2019-02-25

**Authors:** Sang Mi An, Seulgi Kwon, Jung Hye Hwang, Go Eun Yu, Deok Gyeong Kang, Da Hye Park, Tae Wan Kim, Hwa Chun Park, Jeongim Ha, Chul Wook Kim

**Affiliations:** 1 Swine Science and Technology Center, Gyeongnam National University of Science & Technology, Jinju, 52725, South Korea; 2 Dasan Pig Breeding Co., Namwon, 55716, South Korea

## Abstract

In pigs, litter size is typically defined as the total number of piglets born (TNB) or the number of piglets born alive (NBA). Increasing pig litter size is of great economic interest as a means to increase productivity. The capacity of the uterus is a critical component of litter size and may play a central role in prolificacy. In this study, we investigated litter-size-related epigenetic markers in uterine tissue from Berkshire pigs with smaller litter size groups (SLGs) and larger litter size groups (LLGs) using genome-wide bisulfite sequencing (GWBS). A total of 3269 differentially methylated regions (DMRs) were identified: 1566 were hypermethylated and 1703 hypomethylated in LLG compared to SLG. The zona pellucida binding protein (*ZPBP*) gene was significantly hypomethylated in the LLG promoter region, and its expression was significantly upregulated in uterine tissue. Thus, the methylation status of *ZPBP* gene was identified as a potential indicator of litter size. Furthermore, we verified its negative correlation with litter size traits (TNB and NBA) in whole blood samples from 172 Berkshire sows as a blood-based biomarker by a porcine methylation-specific restriction enzyme polymerase chain reaction (PMP) assay. The results suggest that the methylation status of the *ZPBP* gene can serve as a valuable epigenetic biomarker for hyperprolific sows.

## 1 Introduction

In commercial pig farming, increasing litter size is of great economic interest as a means to increase production (Balcells et al., 2011; Nielsen et al., 2013; Rutherford et al., 2013). Litter size is typically defined as the total number of piglets born (TNB) or the number of piglets born alive (NBA). Litter size is controlled by many factors, such as ovulation rate (number of ovulated eggs), number of corpora lutea, fertilization rate, uterine capacity, and prenatal survival (Distl, 2007; Mesa et al., 2003). Fetal survival is primarily determined by the uterine capacity of the dam, which can be defined in terms of the relative surface area of placental endometrial attachment required to support the nutrient requirements of an individual fetus throughout gestation (Wilson et al., 1998). Therefore, uterine traits can greatly influence litter size. In addition, the low heritability of litter size (5 %–10 %) suggests that proximate environmental variables may contribute significantly to variation in it (Dube et al., 2012).

DNA methylation is among the main epigenetic mechanisms and plays significant roles in gene silencing (Newell-Price et al., 2000), tissue differentiation (Laurent et al., 2010), cellular development (Smith and Meissner, 2013), X-chromosome inactivation (Pollex and Heard, 2012), and genetic imprinting (Li et al., 1993). Importantly, DNA methylation is both stably heritable and fully reversible. DNA methylation may reflect interactions between genetic and environmental factors in the development and reproduction of pigs. In particular, when DNA methylation occurs in a gene promoter, it typically acts to repress gene transcription. Several studies have suggested a correlation between differentially methylated regions (DMRs) near promoter regions and gene expression changes (Lister et al., 2009; Meissner et al., 2008; Varley et al., 2013). Furthermore, these types of dynamic changes in DNA methylation tend to occur during embryonic development as cells differentiate or become reprogrammed (Hajkova et al., 2002; Mayer et al., 2000; Sasaki and Matsui, 2008). Many recent studies have examined the genome-wide methylation profiles of livestock phenotypes associated with disease resistance, milk production, and reproduction (Congras et al., 2014; Coster et al., 2012; Jin et al., 2014; Singh et al., 2012). DNA methylation affects the expression of many genes that are critical to reproductive traits (Calicchio et al., 2014; Messerschmidt et al., 2014; Stevenson and Prendergast, 2013). Hwang et al. (2017) recently identified DMRs and differentially expressed genes (DEGs) associated with litter size in pig placentas and suggested that the *PRKG2*, *CLCA4*, and *PCK1* genes play important roles in improving litter size by increasing nutrition supply through the placenta.

The objective of this study was to use epigenetic approaches to examine uterine tissues of Berkshire pigs with smaller and larger litter sizes, using genome-wide bisulfite sequencing (GWBS) technology. Our findings will provide useful knowledge and a clearer understanding of the reproductive phenotypes of individual pigs and could help in selecting sows with high fecundity for breeding.

**Figure 1:**
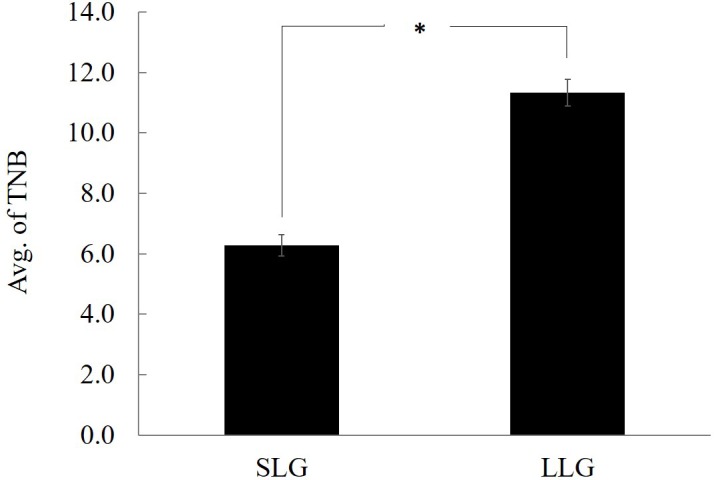
Comparison of average of TNB between SLG and LLG. The bars represent the mean ± SD; n=3. ${}^{*}$ Significantly different between both groups at p<0.05. TNB: total number of piglets born; SLG: smaller litter size group; LLG: larger litter size group.

## 2 Materials and methods

### 2.1 Animals and sample preparation

All animal experiments were approved by the Gyeongnam National University of Science and Technology Institutional Animal Care and Use Committee (permit no. 2105-5). All Berkshire sows used in this study were reared under the same environmental conditions (Dasan Pig Breeding Co., Namwon, Korea) and provided with the same commercial diet and water ad libitum. To analyze DNA profiling according to litter size, animals were divided into smaller litter size groups (SLGs; average litter size ≤7) and larger litter size groups (LLGs; average litter size ≥10), and three sows were randomly selected from each group (Fig. 1). The uterus was collected immediately after slaughter and flushed with phosphate-buffered saline (PBS); the endometrium was then separated from the uterus. The collected endometrium was rapidly frozen in liquid nitrogen and stored at -80 ${}^{\circ }$C until further analysis. To verify the usefulness of the candidate gene as a prognostic tool, whole blood samples were collected from 172 sows using BD Vacutainer K${}_{2}$ EDTA tubes (BD, Oxford, UK) as an anticoagulant. Blood was mixed immediately after the draw by inverting the tubes 10 times and then stored at 2–8 ${}^{\circ }$C until use.

### 2.2 DNA methylation

#### 2.2.1 Genome-wide bisulfite sequencing for marker selection

Genomic DNA (gDNA) was isolated from three endometrium tissues per group using a DNeasy Tissue Kit (Qiagen, Valencia, CA, USA) and pooled for GWBS analyses. gDNA (≥6 µg) was fragmented by ultrasonication to approximately 100–300 bps and then end-repaired, 3${}^{\prime }$-end adenylated, and ligated with adapters. Fragmented DNA was bisulfite-converted using the EZ DNA Methylation-Gold Kit (Zymo Research, Orange, CA, USA). Bisulfite-converted DNA was quantified using a Quant-iT dsDNA High Sensitivity Assay Kit (Life Technologies, Rockville, MD, USA) on an Agilent 2100 Bioanalyzer (Agilent Technologies, Inc., Santa Clara, CA, USA) and used as a template for polymerase chain reaction (PCR) amplification. After quantitative PCR (qPCR) amplification, the resulting libraries were subjected to paired-end sequencing with a 100 bp read length using the Illumina HiSeq 2500 platform (Illumina, San Diego, CA, USA). The raw sequencing reads were cleaned by removing adaptor sequences, and reads with a percentage of unknown bases greater than 10 % and low-quality reads (more than 20 % of < Q20 bases) were filtered out to retain only high-quality reads. Clean reads were then mapped to the pig reference genome (Sscrofa 10.2) using Bismark software (version 0.9.0) with two allowed mismatches (Krueger and Andrews, 2011). Methylated cytosines were extracted from aligned reads using the Bismark methylation extractor with standard parameters. The methylation level of a cytosine (C) within an aligned read was determined by calculating the ratio of the number of reads containing a methylated C at the location to the number of all reads covering the location. DMRs between the two groups were predicted using CpG_MP with the default parameters (length, cytosine–guanine (CG) content, and cytosine : phosphate : guanine (CpG) ratio) (Su et al., 2013). We identified differentially methylated genes (DMGs) when a DMR and specific gene function element (e.g., a promoter) overlapped in the University of California Santa Cruz Genome Browser Database. Gene Ontology (GO) analysis was performed for gene functional annotation using DAVID Bioinformatics Resources (version 6.7; http://david.abcc.ncifcrf.gov/, last access: 13 April 2018).

**Table 1:** Primer sequences used for PMP assay and RT-PCR.

Application	Gene	Accession no.	Primer (5${}^{\prime }\to 3^{\prime }$)	Product size
PMP assay	*ZPBP*	NC_010451.3	F: TCAGGTGAGGCGTCGGCAT	162
			R: CGTCATCAATGTCCAGTCCT	
RT-PCR	*ZPBP*	NM_214106.1	F: CTGGATTAACCGCTGCTTTC	158
			R: ATGCTTTTGCTCCAAACACC	
	*PPIA*	NM_214353.1	F: CACAAACGGTTCCCAGTTTT	171
			R: TGTCCACAGTCAGCAATGGT	

F: forward; R: reverse.

#### 2.2.2 Porcine methylation-specific restriction enzyme PCR assay

The Porcine methylation-specific restriction enzyme PCR (PMP) assay is a PCR-based methylation method that uses methylation-sensitive restriction enzymes to determine DNA methylation status. To verify the result of GWBS, whole blood samples were collected from Berkshire sows to isolate gDNA using a Wizard Genomic DNA Purification Kit (Promega, Madison, WI, USA) according to the manufacturer's instructions and digested with *Hpa*II (NEB, Hitchin, UK) and *Msp*I (NEB), a pair of methylation-sensitive isoschizomers that have the same recognition site (CC|GG). An undigested gDNA (5 µg) served as the negative control. Gene-specific primers were designed to flank the *Hpa*II/*Msp*I sites; these are described in Table 1. PCR was performed under the following conditions: 94 ${}^{\circ }$C for 5 min, followed by 35 cycles of 94 ${}^{\circ }$C for 30 s, 60 ${}^{\circ }$C for 30 s, and 72 ${}^{\circ }$C for 30 s. The products were electrophoresed on a 2 % (w/v) agarose gel in 6X loading buffer (Biosesang, Seongnam, Korea).

### 2.3 Reverse-transcription PCR analysis

Reverse-transcription PCR (RT-PCR) analysis was performed to detect gene expression. Total RNA was extracted from three uterine tissues from each group using the TRIzol Reagent (Molecular Research Center, Cincinnati, OH, USA) and then reverse-transcribed into cDNA using Superscript II Reverse Transcriptase (Invitrogen, Carlsbad, CA, USA). cDNA was then subjected to RT-PCR to evaluate the relative gene expression levels of the zona pellucida binding protein (*ZPBP*) and the gene encoding peptidylprolyl isomerase A (*PPIA*) (internal control), using appropriate primer pairs (Table 1). Amplification was performed using a Perkin Elmer 9700 system (Applied Biosystems, Waltham, MA, USA) under the following conditions: 95 ${}^{\circ }$C for 5 min; 30 cycles of 95 ${}^{\circ }$C for 30 s, 60 ${}^{\circ }$C for 30 s, 72 ${}^{\circ }$C for 30 s, and final elongation for 7 min at 72 ${}^{\circ }$C. The amplification products were separated on 2 % (w/v) agarose gel and quantified using a Gel Logic model 200 imaging system (Kodak, Rochester, NY, USA).

### 2.4 Statistical analysis

Comparisons between groups were performed using t tests, with statistical significance determined at p<0.05. The results are expressed as means ± standard deviation (SD). Linear regression analyses were used to test the relationships between *ZPBP* methylation status and sow litter size traits (TNB and NBA). All statistical analyses were conducted using SPSS software (version 20.0; SPSS Inc., Chicago, IL, USA).

**Table 2:** Summary of sequencing results and reads alignment.

Group	SLG	LLG
Raw reads (no.)	1 248 683 696	1 217 238 456
(Read depth, X)	41.62 X	40.57 X
Analyzed reads (no.)	1 107 209 686	1 076 828 732
(%)	(88.67 %)	(88.46 %)
Mapped reads (no.)	662 804 470	614 402 588
(%)	(53.08 %)	(50.475 %)
Uniquely mapped reads (no.)	594 374 396	549 444 152
(%)	(47.60 %)	(45.14 %)

## 3 Results and discussion

### 3.1 Identification of the *ZPBP* gene as an epigenetic marker

GWBS was performed on gDNA from pooled uterus samples (n=3 for both SLG and LLG). In total, 1249 and 1217 million raw reads were generated in SLG and LLG, respectively. The mapped reads covered 53.08 % (SLG) and 50.48 % (LLG) of the pig genome (Table 2). A total of 3269 DMRs were discovered; 1566 were hypermethylated and 1703 were hypomethylated in LLG compared to SLG (Table 3). The DMRs were determined by considering the p value (<0.01) and false discovery rate (Q value <0.01). Among genes with DMRs, *ZPBP* was found to be strongly related to fecundity by GO enrichment analysis. This DMG was mainly observed at promoter regions (UP1kb; 1 kb region upstream of transcription start sites) and was hypomethylated in LLG (Table 4).

**Table 3:** Numbers and ratio of hyper-DMRs and hypo-DMRs.

Sample	Hyper-DMRs	Hypo-DMRs	Total number
			of DMRs
SLG vs. LLG	1566 (47.9 %)	1703 (52.1 %)	3269

DNA methylation plays an important role in regulating gene expression (Wen et al., 2016). In particular, DNA methylation in gene promoters is strongly associated with gene silencing (Bell et al., 2011; Lande-Diner and Cedar, 2005; Weber et al., 2007). Recently, studies have been conducted to identify the genome-wide methylation profiles of farm animals (Hao et al., 2016; Hu et al., 2013; Xu et al., 2016; Zhang et al., 2014). Some studies have described DNA methylation for the pig uterus (Bartol et al., 2008; Franczak et al., 2017; Ko et al., 2008; Pistek et al., 2013); however, few have reported uterine genome-wide methylation patterns. GWBS, which allows unbiased genome-wide DNA methylation profiling, has been used to investigate prolificacy-related DNA methylation in unprecedented detail (Kurdyukov and Bullock, 2016). In the current study, we used GWBS to investigate the DNA methylation profiles of the genome in uterine tissues of high- and low-prolificacy pigs to explore the relationships between DNA methylation and litter size traits.

**Table 4:** Information of DNA methylation of *ZPBP*.

Gene	Chr	Start	End	DMR	Log	Difference	Pattern	p value	FDR
				position	(L/S)	(S-L; cutoff 0.2)		(p<0.01)	(Q<0.01)
*ZPBP*	Chr 9	149 712 887	149 713 279	UP1kb	-101419348	0.22456	Hypo	$1.32\times 10^{-14}$	$1.89\times 10^{-11}$

FDR: false discovery rate; S: smaller litter size group; L: larger litter size group.

**Figure 2:**
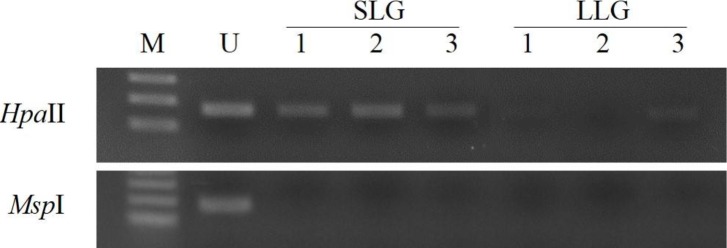
Methylation analysis of the *ZPBP* gene in whole blood samples of Berkshire sows by PMP assay. gDNAs were cut with the methylation-sensitive restriction enzymes *Hpa*II/*Msp*I. M: size marker; U: undigested DNA; SLG: smaller litter size group; LLG: larger litter size group.

### 3.2 Confirmation of the methylation status of the *ZPBP* gene in whole blood by PMP assay

To assess the prognostic capability of the *ZPBP* gene as a blood-based biomarker for increased litter size, DNA methylation patterns of the *ZPBP* gene were verified in whole blood samples collected from three sows, used to obtain uterus tissues in each group by PMP assay. As shown in Fig. 2, we confirmed that the *ZPBP* gene was hypomethylated in LLG. This result was consistent with the results of GWBS analysis, and will allow early prediction of litter sizes in prepubertal gilts from blood samples without slaughter.

### 3.3 Gene expression of hypomethylated *ZPBP* in the promoter region

Next, to investigate the gene expression of *ZPBP* with hypomethylated promoter, RT-PCR was performed on uterine tissues of three Berkshire sows from each group. As shown in Fig. 4, *ZPBP* gene expression was significantly upregulated in LLG, as expected. Promoter methylation generally impedes the binding of transcription factors and in a second stage leads to chromatin condensation, with long-term repression of gene expression (Schubeler, 2015). Therefore, this result supports the hypothesis that *ZPBP* overexpression in uterine tissue is due to promoter hypomethylation.

**Figure 3:**
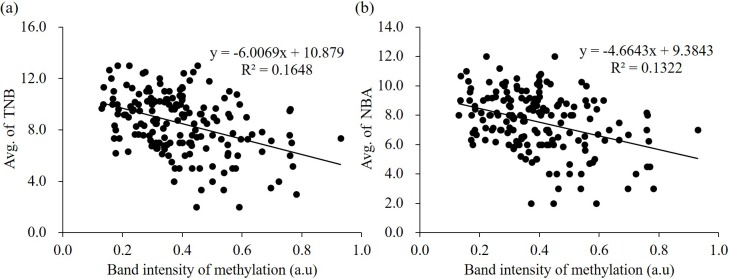
Correlation between methylation status of *ZPBP* gene and litter size traits (TNB and NBA) in 172 Berkshire sows using logistic regression analysis. The band intensities of PMP assay are shown on the x axis, and the y axis shows TNB **(a)** and NBA **(b)**. TNB: total number of piglets born; NBA: number of piglets born alive: a.u: arbitrary unit.

As the DNA methylation status of promoter regions could affect gene expression through changes in chromatin structure or transcription efficiency (Klose and Bird, 2006; Lorincz et al., 2004), we compared the genome-wide methylation patterns of high- and low-prolificacy pigs to identify DMGs that might affect prolificacy traits such as litter size.

**Figure 4:**
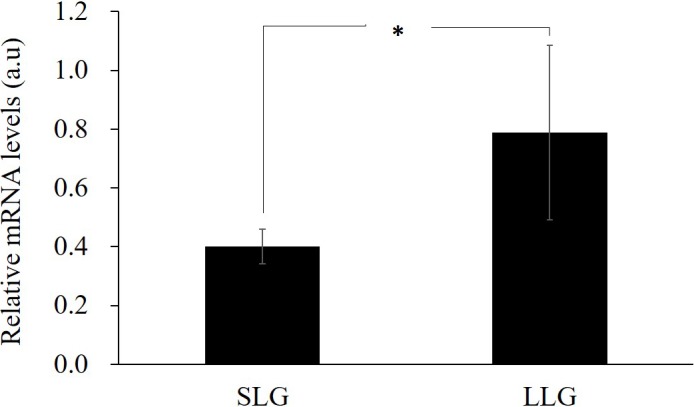
The effect on gene expression of promoter hypomethylated *ZPBP* in uterine tissues by RT-PCR. The bars represent the mean ± SD; n=3. ${}^{*}$ Significantly different between both groups at p<0.05. SLG: smaller litter size group; LLG: larger litter size group.

### 3.4 Verification of the relationship between methylation status of the *ZPBP* gene and litter size traits

We verified the relationship between methylation status of the *ZPBP* gene and litter size traits in Berkshire sows (n=172) using a PMP assay (Fig. 4). The amplified products were normalized using undigested DNA samples. To determine the relationship, linear regression analyses were performed; the methylation status of the *ZPBP* gene exhibited negative relationships with litter size traits. Logistic regression analyses of the results of TNB ($R^{2}=0.1648$) indicated the following: TNB (y)=-6.0069×ZPBP(x)+10.879 (Fig. 3a). When NBA was included in the model, the relationship was as follows: $R^{2}=0.1322$; NBA (y)=-4.6643×ZPBP(x)+9.3843 (Fig. 3b). These results confirm that *ZPBP* methylation status was significantly negatively associated with litter size traits (TNB and NBA) and that sows with hypomethylated *ZPBP* had high fecundity. Thus, the *ZPBP* gene can act as an epigenetic marker for early prediction of litter size in Berkshire pigs.

Zona pellucida (ZP) is a filamentous matrix of well-structured and glycosylated glycoproteins surrounding the oocyte that acts as a morphological criterion for oocyte selection. This matrix is formed of three proteins encoded by three different genes: ZP1, ZP2, and ZP3 (Pokkyla et al., 2011; Wassarman, 2008). *ZPBP*, which localizes to the acrosomal membrane and likely interacts with multiple acrosomal matrix proteins, was named for its function, binding to the oocyte ZP protein following the acrosome reaction (Yu et al., 2009). *ZPBP* mainly acts in acrosome compaction and sperm morphogenesis during spermiogenesis. Most research on *ZPBP* has focused on its location in the acrosome of the sperm and its function in sperm–oocyte interactions during fertilization (Mori et al., 1993, 1995; Yu et al., 2006). However, Campbell et al. (2006) reported that murine *ZPBP* was a luminal epithelium-specific gene with 20-fold or higher expression in the uterine luminal epithelium than in the stroma–glandular epithelium. Implantation is essential for the establishment of normal pregnancy and is initiated by a physical interaction between the trophoblast and the apical surface of the luminal epithelium, followed rapidly by adhesion and then by penetration through the luminal epithelium to the underlying stroma, which responds by decidualization (Abrahamsohn and Zorn, 1993). In other words, the uterine luminal epithelium plays a critical role in implantation. *ZPBP* is a serine protease (serine protease 38) with ZP-binding properties that were initially identified in porcine epididymal sperm (Mori et al., 1993). Serine proteases are characterized by the presence of serine as a nucleophilic amino acid at the active site of the enzyme (Hedstrom, 2002). Some serine proteases are detectable in the uterus and are involved in female reproduction, especially in oocyte development, ovulation, implantation, and decidualization (Diao et al., 2013; Nie et al., 2005). Therefore, we believe that *ZPBP* might play a role in female reproduction, including implantation.

## 4 Conclusions

This study reports the DNA methylation patterns of porcine uterine tissues, which are associated with litter size. We identified DMRs and detected hypomethylation of the *ZPBP* gene in its promoter region in LLG; its expression was upregulated in uterine tissues. We also verified that the methylation status of the *ZPBP* gene was significantly negatively associated with litter size traits in the larger pig population. Our results demonstrate that this gene can be used as a biomarker for hyperprolific sows and will likely contribute to improving reproductive capacity.

## Data Availability

The data sets are available upon request from the corresponding author.
